# Predictors of caregiver burden before starting family-based treatment for adolescent anorexia nervosa and associations with weight gain during treatment

**DOI:** 10.1007/s40519-023-01553-4

**Published:** 2023-02-21

**Authors:** Abigail Matthews, Amanda B. Bruening, Claire M. Aarnio-Peterson, Rachel Kramer

**Affiliations:** 1 Department of Pediatrics, University of Cincinnati College of Medicine, Cincinnati, OH USA; 2 Division of Behavioral Medicine and Clinical Psychology, Cincinnati Children’s Hospital Medical Center, Cincinnati, OH USA; 3 Department of Psychiatry and Behavioral Sciences, University of California, San Francisco, San Francisco, CA USA

**Keywords:** Anorexia nervosa, Adolescents, Caregiver burden, Family-based treatment

## Abstract

**Purpose:**

Caregivers play a pivotal role in the success of family-based treatment (FBT) for anorexia nervosa (AN). Caregiver burden is frequently demonstrated in eating disorders (EDs) and may impact FBT outcomes. This study examined factors associated with caregiver burden before starting FBT and whether pre-treatment caregiver burden was associated with weight gain during FBT.

**Methods:**

Participants included 114 adolescents with AN or atypical AN (mean age = 15.6 years, SD = 1.4) and a primary caregiver (87.6% mothers) who received FBT in the United States. Before starting treatment, participants completed self-report measures of caregiver burden (via the Eating Disorder Symptom Impact Scale), caregiver anxiety, caregiver depression, and ED symptoms. Clinical characteristics and percentage of target goal weight (%TGW) at FBT session 1 and 3 and 6 months after starting treatment were obtained via retrospective chart review. Hierarchical regressions examined predictors of caregiver burden before FBT initiation. Associations between pre-treatment caregiver burden and %TGW gain at 3 and 6 months after starting FBT were assessed with hierarchical regressions.

**Results:**

Caregiver anxiety (*p* < 0.001), family history of EDs (*p* = 0.028), adolescent mental health treatment history (*p* = 0.024), and ED symptoms (*p* = 0.042) predicted caregiver burden before starting FBT. Pre-treatment caregiver burden was not associated with %TGW gain at 3 or 6 months. Males demonstrated less %TGW gain than females at 3 months (p = 0.010) and 6 months (*p* = 0.012).

**Conclusion:**

Proactively evaluating caregiver burden before starting FBT is suggested. Providing recommendations and/or referrals for identified caregiver vulnerabilities could indirectly impact FBT progress. Males in FBT could require longer courses of treatment and extra vigilance to this demographic is suggested.

*Level of evidence:* Level III, case–control analytic study.

Family-based treatment (FBT) [1] has the greatest evidence-base in the treatment of adolescents with anorexia nervosa (AN) and has demonstrated faster recoveries, higher sustained remission rates, and fewer hospitalizations [2–4] than other forms of psychotherapy. Hallmark characteristics of AN such as intense fear of gaining weight, distorted body perceptions, and poor insight can preclude adolescents from independently engaging in treatment and gaining necessary weight. As such, caregivers in FBT are considered the active agents of change in recovery and assume control over the adolescent’s eating behaviors until weight is restored.

Research suggests that caregivers play a key role in the success of FBT. For example, caregivers’ beliefs about and behaviors about their adolescent’s eating disorder (ED) are associated with illness course and outcomes [5]. Parent self-efficacy, or a caregiver’s confidence in their ability to help the adolescent overcome AN, is positively associated with weight gain in FBT [6, 7]. Conversely, higher expressed emotion, or parent criticism, hostility, and emotional overinvolvement, is related to treatment dropout [8] and ED symptom severity [9]. Caregiver anxiety and depression are also associated with poorer treatment outcomes [10].

Caregiver burden, or a caregiver’s physical, psychological, financial, and/or social strain secondary to the sustained efforts of caring for someone with a chronic illness, is common among ED caregivers [11]. Significant associations between caregiver burden, parent self-efficacy, and expressed emotion [12, 13] have been demonstrated, suggesting a possible association between caregiver burden and FBT outcomes. However, few studies have examined caregiver burden in FBT. Across diverse ED treatment samples, greater caregiver burden has been associated with caregiver anxiety and depression [14], limited social support [15, 16], maladaptive coping styles [15], negative beliefs about EDs [17], divorced marital status [14], being a female caregiver [18], having a personal or family history of EDs [19], and spending more time in caregiving tasks [20, 21].

FBT is generally deemed the most efficacious treatment for adolescent AN, yet more than 50% of adolescents may not achieve full remission in this approach [22]. It is crucial to identify factors associated with FBT outcomes to develop targeted, adjunctive interventions to boost treatment efficacy. High rates of caregiver burden in EDs [16, 21, 23, 24], coupled with previously demonstrated associations between caregiver characteristics and treatment outcomes [5, 25, 26], suggest that caregiver burden could impact FBT success. Our study aimed to describe factors associated with caregiver burden before starting FBT and to evaluate the association between caregiver burden and adolescent weight gain over the course of FBT.

## Methods

### Participants and procedure

Through retrospective chart review, 114 adolescents (aged 12–18) who were diagnosed with AN (*n* = 54, 47.4%) or atypical anorexia nervosa (AAN; *n* = 60, 52.6%) between July 2017 and April 2022 were identified. Adolescents received outpatient FBT within a multidisciplinary ED program at a pediatric academic medical center in the Midwestern United States.

Adolescents seeking specialized care at our institutional ED program are initially evaluated by an adolescent medicine physician. At that visit, adolescents diagnosed with AN/AAN who are medically stable are referred to outpatient FBT if clinically indicated. Individuals with AN/AAN and acute medical complications upon presentation (e.g., bradycardia, electrolyte imbalance, risk for refeeding syndrome) are emergently hospitalized on an adolescent medicine unit within the institution. Most inpatients with newly diagnosed AN/AAN are discharged home, to outpatient FBT within the multidisciplinary ED program. Caregivers of inpatients receive a brief FBT-guided intervention [27] during their adolescent’s admission, including psychoeducation about AN, including the need for caregivers to assume control over the adolescent’s eating behaviors at home. Notably, hospitalized patients with imminent safety concerns and/or complex psychiatric comorbidities are transferred to inpatient psychiatric care upon medical stabilization. These patients were excluded from our study.

Prior to the first outpatient FBT session, adolescents and their primary caregivers complete self-report questionnaires to guide clinical conceptualization and treatment recommendations. Questionnaires are completed electronically at home or, for patients who are hospitalized prior to the first outpatient FBT session, in the hospital within the first 72 h of admission. Outpatient FBT is conducted by an FBT-trained psychologist, postdoctoral psychology fellow, or master’s level clinician within the ED program. Outpatients also receive medical management from an adolescent medicine physician, and per needed, visits with a specialized dietitian over the course of FBT. Patient weights are measured at outpatient visits across disciplines.

Adolescents with AN/AAN were excluded from our study if they and/or their caregiver were not fluent in English. Among medically hospitalized patients, those transferred to inpatient psychiatric care upon stabilization (versus discharging home) were also excluded. Adolescents and caregivers provided assent and consent, respectively, for inclusion of questionnaires and prospective medical data in a large research repository. This study was approved by the Institutional Review Board.

## Measures

### Self-report questionnaires

#### Caregiver burden

Caregivers completed the Eating Disorder Symptom Impact Scale (EDSIS) [28], a 24-item self-report measure of burden in ED caregivers. EDSIS items assess the frequency of negative behaviors (“Were there arguments or tensions during mealtimes?”) and aversive emotional states (“Feeling that there could have been something I should have done.”) experienced in the last month. Items are rated on a 5-point Likert scale ranging from never (0) to nearly always (4). EDSIS total scores were used in analyses, with higher scores representing more caregiver burden. The EDSIS demonstrates acceptable convergent validity (*r* = 0.30–0.60) and good internal consistency (*α* = 0.84–0.90) [28]. Internal consistency was good in our sample (*α* = 0.86).

#### Caregiver mental health

Caregiver symptoms of anxiety and depression were assessed with the Generalized Anxiety Disorder-7 (GAD-7) [29] and the Patient Health Questionnaire-8 (PHQ-8) [30, 31]. The GAD-7 is a self-report assessment of anxiety symptoms experienced in the last 2 weeks. Seven items are answered on a 4-point Likert scale, ranging from not at all (0) to nearly every day (3) and are summed into a severity index, with higher scores reflecting greater anxiety. The GAD-7 demonstrates adequate sensitivity (0.89) and specificity (0.82) when using a clinical cutoff score of 10, excellent internal consistency (*α* = 0.92) and good test–retest reliability (*r* = 0.83), and excellent internal and external validity [29]. The PHQ-8 measures depressive symptoms in the past two weeks, with items reflecting DSM-5 diagnostic criterion of depression [32], except suicidality. The original PHQ-9 [30] includes a suicide item, yet we use the PHQ-8 because questionnaires are completed electronically and off-site, prohibiting real-time intervention for those with imminent safety concerns. PHQ-8 items are answered with the same 4-point Likert scale used in the GAD-7 and are totaled into a severity index, with higher scores indicating more severe depressive symptoms. A clinical cutoff score of 10 has been demonstrated for the PHQ-9 [30], and research shows nearly identical scoring thresholds and accuracy for the PHQ-8 [33]. The PHQ-9 is a reliable (*α* = 0.86–0.89) and valid measure of depressive symptoms in adult samples [30, 31]. Good internal consistency was demonstrated for the GAD-7 (*α* = 0.87) and the PHQ-8 (*α* = 0.87) in our sample.

#### Eating disorder symptoms

Adolescents completed the Eating Disorder Examination-Questionnaire (EDE-Q) [34], a 28-item, self-report assessment of ED attitudes and behaviors over the past 28 days. The EDE-Q Global scale was used in analyses, with higher scores indicating more severe ED symptoms. The EDE-Q demonstrates good internal consistency (*α* = 0.70–0.93), criterion validity, and construct validity across studies [35]. Excellent internal consistency was demonstrated for the EDE-Q Global scale in our sample (*α* = 0.96).

### Demographic questionnaires

Adolescents self-reported gender identity, race, and sexual orientation. Caregivers self-reported marital status, relationship to the adolescent, adolescent history of mental health treatment (prior to AN development), and family history of EDs.

### Medical data

Medical data were obtained retrospectively, via electronic medical records. Extracted data included adolescent age, diagnosis (AN or AAN), illness duration (months), inpatient hospitalization status before starting outpatient FBT (yes or no). Premorbid body mass index percentile for age-and-sex (BMI%ile) was determined by reviewing historical growth trends, prior to the onset of weight loss. For adolescents without a clear premorbid BMI%ile trend, the median of all recorded, historical BMI%ile values was calculated.

Adolescent weights were extracted from multidisciplinary ED clinic visits that occurred at four time points: (1) the date that self-report questionnaires were completed; (2) the date of the first outpatient FBT session (i.e., “FBT session 1”); (3) 3 months after FBT session 1; and (4) 6 months after FBT session 1.

Patient-specific treatment goal weights (TGWs) were calculated for each time point, based on premorbid growth trends [36]. We used TGW as a metric instead of median body weight (i.e., expected weight at the 50^th^ BMI%ile for age-and-sex) because 87.6% of our sample had a premorbid BMI%ile for age-and-sex above the 50th (mean premorbid BMI%ile = 70.0, SD = 24.0). TGWs were calculated by research team members to ensure a standardized calculation methodology. Next, percentage of TGW (%TGW) was calculated for each timepoint (baseline, FBT session 1, 3 months after starting FBT, 6 months after starting FBT) by dividing the patient’s recorded weight at that timepoint by TGW at that timepoint and multiplying by 100. Change in %TGW (i.e., %TGW gain) at 3 and 6 months after starting FBT was also calculated, including %TGW gain at 3 months (%TGW at 3 months *minus* %TGW at FBT session 1) and %TGW gain at 6 months (%TGW at 6 months *minus* %TGW at FBT session 1).

### Statistical analyses

Clinical and demographic characteristics for adolescents and caregivers in our sample were described using SPSS version 27, including adolescent age, gender identity, sexual orientation, race, diagnosis, illness duration, hospitalization before starting FBT, family history of EDs, adolescent history of mental health treatment (before AN onset), caregiver type, caregiver marital status, caregiver burden (EDSIS), caregiver anxiety (GAD-7), and caregiver depression (PHQ-8) before starting FBT, adolescent ED symptoms (EDE-Q) before starting FBT, %TGW at the time of questionnaire completion, %TGW at FBT session 1, %TGW 3 months after starting FBT, and %TGW 6 months after starting FBT. We also assessed the frequency that caregivers met or exceeded defined clinical cut-offs on the GAD-7 and PHQ-8 (i.e., severity indices ≥ 10) [29, 31].

To examine predictors of caregiver burden before starting FBT (aim 1), hierarchical regression analyses were conducted using two steps. Adolescent characteristics were entered in Step 1, including age, gender identity, illness duration, family history of EDs (yes/no), history of mental health treatment (yes/no), %TGW when questionnaires were completed, and ED symptoms (EDE-Q). Step 2 assessed whether caregiver anxiety (GAD-7), caregiver depression (PHQ-8), and caregiver marital status (married/single) predicted caregiver burden (EDSIS) over and above adolescent characteristics. Caregiver type was not included in analyses due to low statistical power, with 87.6% of caregivers being mothers.

The association between caregiver burden before starting treatment and %TGW gain at 3 and 6 months after starting FBT (aim 2) was first examined with paired *t*-tests that assessed whether %TGW gain was significant from FBT session 1 to 3 months after starting FBT and from FBT session 1 to 6 months after starting FBT. Next, two-step hierarchical regression analyses assessed whether caregiver factors predicted %TGW gain from FBT session 1 to 3 months and from FBT session 1 to 6 months. Step 1 included factors associated with ED risk and prognosis in previous research studies [19, 37–40], including adolescent age, gender identity, illness duration, %TGW at FBT session 1, hospitalization before starting outpatient FBT (yes/no), and ED symptoms (EDE-Q). Step 2 included caregiver burden (EDSIS), caregiver anxiety (GAD-7), and caregiver depression (PHQ-8).

Fisher–Freeman–Hoffman tests were conducted to assess the association between %TBW at FBT session 1 and achieving weight restoration (≥ 95% TGW) at 3 and 6 months after starting FBT. Patients were divided into three groups based on %TGW at FBT session 1, including *low* (defined by %TGW at FBT session 1 less than 1 SD of the sample mean), *average* (defined by %TGW at FBT session 1 within 1 SD of the sample mean), and *high* (defined by %TGW at FBT session 1 greater than 1 SD of the sample mean). Fischer’s exact tests (given cell counts less than 5) assessed for gender differences in achieving weight restoration (≥ 95% TGW) at 3 and 6 months. Chi Square analyses evaluated whether being medically hospitalized before starting outpatient FBT was significantly associated with achieving weight restoration (≥ 95% TGW) at 3 and 6 months.

Power analyses were conducted using G-Power 3.1 [41] given numerous predictors in the regression models.

## Results

### Demographic and clinical characteristics

The mean age of adolescents in our sample was 15.55 years (SD = 1.36). Most identified as female (*n* = 95, 83.3%), 12.3% identified as male (*n* = 14), and 4.5% identified as transgender or gender non-binary. Most of our sample was White (*n* = 105, 94.6%), 0.9% was Black (*n* = 1), 0.9% was Asian (*n* = 1), and 3.6% identified race as “other” (*n* = 4). Regarding sexual orientation, 80.5% of adolescents in our sample identified as straight (*n* = 91), 6.2% identified as gay or lesbian (*n* = 7), 3.5% identified as asexual (n = 4), and 9.7% reported sexual orientation as “other” (*n* = 11). Most caregivers in our sample were mothers (*n* = 85, 87.6%), 10.3% were fathers (*n* = 10), and 2% reported their relationship to the adolescent as “other” (*n* = 2). Most caregivers were married (*n* = 57, 79.2%), 16.7% were single, divorced, or separated (*n* = 12), and 4.2% reported a marital status as “other” (*n* = 3). Eighty adolescents in our sample (63.5%) were medically hospitalized before starting FBT and 46 (36.5%) were not. Significant differences in patient or caregiver characteristics were not found for patients with medical hospitalization before starting outpatient FBT versus without admissions (all *p*s > 0.050). Twelve adolescents (10.5%) in our sample prematurely terminated FBT (i.e., attended 1 or more FBT session(s) but failed to schedule additional sessions despite therapist recommendations to do so). Significant differences for adolescent or caregiver characteristics were not found among patients who prematurely terminated FBT (all *p*s > 0.050). Additional characteristics of our sample are summarized in Table 1.

**Table 1:** Clinical characteristics of adolescents with AN/AAN and their primary caregivers in FBT

	Total *N* = 114
	*n* (%)
Diagnosis	
AN	54 (47.4)
AAN	60 (52.6)
Medically hospitalized before starting FBT (yes)	75 (65.8)
Past mental health treatment before AN onset (yes)	48 (42.5)
Family history of EDs (yes)	25 (22.3)
	*M* (SD)
Illness duration (months)	10.11 (7.49)
Caregiver burden (EDSIS)	31.12 (12.33)
Caregiver depression (PHQ-8)	5.13 (4.84)
Caregiver anxiety (GAD-7)	4.94 (4.41)
ED symptoms (EDE-Q)	3.41 (1.59)
%TGW at FBT session 1	82.47 (9.10)
%TGW 3 months after starting FBT	87.97 (9.27)
%TGW at 6 months after starting FBT	88.67 (9.95)

*EDSIS* Eating Disorder Symptom Impact Scale, *PHQ-8* Patient Health Questionnaire-8, *GAD-7* Generalized Anxiety Disorder-7, *EDE-Q* Eating Disorders Examination-Questionnaire, %*TGW* percentage of treatment goal weight

### Predictors of caregiver burden before starting FBT

At step 1, hierarchal regression analysis showed that adolescent history of mental health treatment (before developing AN) (*p* = 0.024), family history of EDs (*p* = 0.028), and greater ED symptom severity (*p* = 0.042) were significantly associated with caregiver burden before starting FBT, *F*(7, 107) = 2.36, *p* = 0.028, *R*^2^ = 0.14. Step 2 significantly improved the model, *FChange*(3, 97) = 7.31, *p* < 0.001, *R*^2^ = 0.30, and showed that caregiver anxiety was significantly associated with caregiver burden before starting treatment (GAD-7, *p* < 0.001, Power = 0.99). See Table 2.

**Table 2:** Predictors of caregiver burden (EDSIS) before starting FBT

	Block 1					Block 2				
	*B*	SE	*t*	*p*	95% CI	*B*	SE	*t*	*p*	95% CI
Constant	41.90	16.35	2.56	0.012	9.45, 74.34	40.60	15.01	2.71	0.008	10.81, 70.39
Age	− 1.67	0.93	− 1.26	0.210	− 3.00, 0.67	− 1.21	0.86	− 1.41	0.161	− 2.91, 0.49
Gender	0.19	3.43	0.05	0.957	− 6.61, 6.98	− 0.33	3.17	− 0.10	0.918	− 2.91, 0.49
Illness duration	− 0.21	0.16	− 1.28	0.203	− 0.53, 0.12	− 0.16	0.15	− 1.03	0.308	− 4.46, 0.15
%TGW at baseline^a^	0.003	0.13	0.02	0.982	− 0.25, 0.25	− 0.001	0.12	− 0.01	0.990	− 0.24, 0.23
ED symptoms (EDE-Q)	1.56	0.76	2.06	0.042	0.06, 3.06	1.54	0.71	2.18	0.032	0.14, 2.93
Past mental health treatment	5.04	2.44	2.07	0.041	0.20, 9.87	4.62	2.24	2.06	0.042	0.18, 9.06
Family history of EDs	6.79	2.80	2.43	0.017	1.24, 12.33	4.60	2.62	1.76	0.082	− 0.60, 9.80
Marital status						− 3.48	2.16	− 1.61	0.110	− 7.76, 0.81
Caregiver depression (PHQ-8)						− 0.21	0.31	− 0.69	0.491	− 0.83, 0.40
Caregiver anxiety (GAD-7)						1.22	0.34	3.56	< 0.001	0.54, 1.90

*%TGW* percentage of treatment goal weight*, EDE-Q* Eating Disorders Examination-Questionnaire, *PHQ-8* Patient Health

Questionnaire-8, *GAD-7 *Generalized Anxiety Disorder-7

^a^*Baseline* corresponds with the time of questionnaire completion

### Associations between pre-treatment caregiver burden and %TGW gain at 3 and 6 months

*T*-tests showed significant %TGW gain from FBT session 1 to 3 months after starting FBT (*t*(101) = 8.44, *p* < 0.001, 95% CI [4.12, 6.65], Cohen’s *d* = 0.84, Power = 1.00). %TGW gain was also significant from FBT session 1 to 6 months after starting treatment (*t*(87) = 8.00, *p* < 0.001, 95% CI [4.83, 8.02], Cohen’s *d* = 0.85, Power = 1.00).

At 3 months after starting FBT, hierarchical regression analysis was significant at step 1, *F*(6, 86) = 5.27, *p* < 0.001, *R*^2^ = 0.27, with gender identity (*p* = 0.005), hospitalization before starting FBT (*p* = 0.030), and %TGW at FBT session 1 (*p* < 0.001) significantly associated with %TGW gain at 3 months. Specifically, adolescents who were medically hospitalized before starting outpatient FBT had gained significantly more %TGW at 3 months. Further, males and adolescents who presented with higher %TGW at FBT session 1 experienced significantly less %TGW gain at 3 months (see Table 3) regardless of hospitalization status before starting outpatient FBT. Step 2 was also significant, *F*(9, 83) = 3.54, *p* < 0.001, but did not demonstrate improvement in the model, *FChange*(3, 83) = 0.29, *p* = 0.830, Power = 0.99).

**Table 3:** Predictors of %TGW gain from FBT session 1 to 3 months after starting FBT

	Block 1					Block 2				
	*B*	SE	*t*	*p*	95% CI	*B*	SE	*t*	*p*	95% CI
Constant	24.62	9.90	2.49	0.015	4.94, 44.29	23.39	10.26	2.28	0.025	3.00, 43.79
Age	0.85	0.50	1.69	0.095	− 0.15, 1.85	0.85	0.52	1.64	0.106	− 0.18, 1.88
Gender	− 5.88	2.04	− 2.88	0.005	− 9.93, − 1.82	− 5.74	2.10	− 2.74	0.008	− 9.91, − 1.57
Illness duration	0.08	0.10	0.79	0.432	− 0.12, 0.78	0.09	0.10	0.89	0.374	− 0.11, 0.30
% TGW at FBT session 1	− 0.27	0.08	− 3.41	< 0.001	− 0.43, − 0.11	− 0.27	0.08	− 3.30	0.001	− 0.43, − 0.11
Hospitalization before starting FBT	− 3.38	1.53	− 2.21	0.030	− 6.43, − 0.34	− 3.32	1.61	− 2.06	0.042	− 6.53, − 0.12
ED symptoms (EDE-Q)	− 0.19	0.43	− 0.45	0.653	− 1.04, 0.65	− 0.22	0.45	− 0.48	0.634	− 1.11, 0.68
Caregiver burden (EDSIS)						0.04	0.06	0.71	0.483	− 0.08, 0.16
Caregiver depression (PHQ-8)						− 0.10	0.21	− 0.48	0.634	− 0.52, 0.32
Caregiver anxiety (GAD-7)						− 0.003	0.25	− 0.01	0.990	− 0.50, 0.49

*%TGW* percentage of treatment goal weight*, EDE-Q* Eating Disorders Examination-Questionnaire, *EDSIS* Eating Disorder Symptom Impact Scale, *PHQ-8* Patient Health Questionnaire-8, *GAD-7* Generalized Anxiety Disorder-7

At 6 months after starting FBT, hierarchical regression analysis was significant at step 1, with gender identity (*p* = 0.002) and %TGW at FBT session 1 (*p* < 0.001) significantly associated with %TGW gain at 6 months, *F*(6, 64) = 6.26, *p* < 0.001, *R*^2^ = 0.31. Specifically, males and adolescents with higher %TGWs at FBT session 1 experienced significantly less %TGW gain at 6 months. Step 2 was also significant, *F*(9, 61) = 5.22, *p* < 0.001, but did not contribute further to the regression, *FChange* (3, 61) = 2.35, *p* = 0.081, Power = 0.99. See Table 4.

**Table 4:** Predictors of %TGW gain from FBT session 1 to 6 months after starting FBT

	Block 1					Block 2				
	*B*	SE	*t*	*p*	95% CI	*B*	SE	*t*	*p*	95% CI
Constant	54.05	12.03	4.49	< 0.001	30.03, 78.08	51.27	12.04	4.26	< 0.001	27.20, 75.35
Age	0.67	0.60	1.13	0.264	− 0.52, 1.87	0.74	0.60	1.13	0.224	− 0.46, 1.95
Gender	− 8.43	2.59	− 3.26	0.002	− 13.60, − 3.26	− 8.31	2.55	− 3.27	0.002	− 13.40, − 3.22
Illness duration	− 0.15	0.12	− 1.33	0.187	− 0.38, 0.08	− 0.10	0.12	− 0.87	0.388	− 0.33, 0.13
%TGW at FBT session 1	− 0.55	0.10	− 5.36	< 0.001	− 0.76, − 0.35	− 0.56	0.10	− 5.53	< 0.001	− 0.77, − 0.36
Hospitalization before starting FBT	− 1.51	1.92	− 0.78	0.436	− 5.35, 2.33	− 0.58	1.97	− 0.30	0.768	− 4.52, 3.35
ED symptoms (EDE-Q)	− 0.12	0.55	− 0.22	0.824	− 1.22, 0.98	− 0.19	0.58	− 0.21	0.751	− 1.35, 0.98
Caregiver burden (EDSIS)						0.10	0.08	1.30	0.200	− 0.06, 0.26
Caregiver depression (PHQ-8)						− 0.29	0.13	− 1.62	0.111	− 0.88, 0.09
Caregiver anxiety (GAD-7)						− 0.08	0.22	− 0.24	0.815	− 0.73, 0.58

*%TGW* percentage of treatment goal weight*, EDE-Q* Eating Disorders Examination Questionnaire, *EDSIS* Eating Disorder Symptom Impact Scale, *PHQ-8* Patient Health Questionnaire-8, *GAD-7* Generalized Anxiety Disorder-7

Fisher–Freeman–Halton exact tests showed that a significantly greater prevalence of patients in the *high* %TGW group at the start of FBT were weight restored (≥ 95% TGW) at 3 months (*n* = 2, 40% of group) and at 6 months (*n* = 3, 60% of group) compared to patients with *low* %TGW (3 months, *n* = 2, 6.7% of group and 6 months, *n* = 3, 11.5% of group) or *average* %TGW (3 months, *n* = 19, 28.8% of group and 6 months, *n* = 29, 33.9% of group). Fisher’s exact tests did not show significant gender differences in achieving weight restoration at 3 months (*p* = 1.00) or 6 months (*p* = 0.497). Being medically hospitalized before starting outpatient FBT was not significantly associated with achieving weight restoration at 3 months, *χ*^2^(1) = 0.25, *p* = 0.805, or 6 months, *χ*^2^(1) = 3.50, *p* = 0.080.

## Discussion

Our study is one of few to examine factors associated with caregiver burden in a large FBT-seeking sample of adolescents with AN/AAN. Consistent with prior findings, a greater prevalence of caregivers in our sample met clinical cut-offs on measures of anxiety and depression (15.2% and 18.6%, respectively) than those reported in community samples of adults (5 and 9%) [31, 42]. Whereas caregiver anxiety significantly predicted pre-treatment caregiver burden as previously demonstrated [14], caregiver depression was not associated with caregiver burden. Inconsistent with past research [14], our finding could reflect lower rates of caregiver depression in our sample compared to those previously reported (19 vs. 38%) [16].

Family history of EDs also predicted caregiver burden, replicating prior findings [19]. Although speculative, having lived experience with an ED, either personally or in a family member, could prime negative beliefs about AN, which have been associated with greater caregiver burden in past studies [17]. We also found that past mental health treatment (before developing AN) and higher EDEQ scores (i.e., greater ED severity) predicted more caregiver burden before starting treatment. Adolescents with past mental health treatment may experience more psychiatric comorbidities that result in more physical, emotional, and financial strain among caregivers. Similarly, greater severity of AN symptoms could necessitate more caregiver resources that perpetuate experiences of burden. Notably, prior studies suggest that mental health comorbidity among adolescents with AN is associated with heightened illness complexity and severity, longer FBT course, and poorer outcomes [8–10, 37, 39].

Significant associations between pre-treatment caregiver burden and %TGW gain at 3 and 6 months after starting FBT were not demonstrated in our study. Conceivably, caregiver burden, at least to a moderate degree, could motivate rather than hinder weight restoration in FBT. At FBT outset, refeeding in AN is conceptualized as a life-or-death decision and caregivers are urged to prioritize their adolescent’s weight gain [1]. These messages undoubtedly increase caregiver distress but are considered necessary to motivate caregiver alignment with FBT. Despite presenting obstacles, treatment purports that caregivers are the key resource in recovery [1].

Our study was limited by the singular assessment of caregiver burden, before starting FBT. Caregiver burden is not static. It is plausible that the severity of caregiver burden *changed* during FBT, and that *changes* in caregiver burden may better predict weight-related outcomes. Parent skills-based interventions are associated with reduced caregiver burden in some studies [43, 44], and FBT participation could indirectly ameliorate burden, thus reducing potential effects on weight restoration. This is evident in previous studies illustrating parent self-efficacy as a mediator of FBT outcomes [45], with positive *change* in parent self-efficacy associated with greater weight gain during treatment. Future studies using a prospective, longitudinal design are warranted to examine the association between *changes* in caregiver burden and weight gain during FBT. Alternatively, the *expression* of caregiver burden (e.g., heightened expressed emotion, accommodation of illness behaviors) versus burden *severity* could impact FBT outcomes and future studies should assess this hypothesis.

Caregiver burden was not associated with marital status, unlike past research [14]. It could be that the relationship between burden and marital status is influenced by other factors that impact burden, like social support and adaptive coping [15], and these variables were not assessed in our study. Further investigation is warranted because non-intact families may require a longer course of FBT [46]. Also inconsistent with past research [14], caregiver burden was not associated with caregiver type. However, statistical power to detect differences was limited, and findings should be interpreted as such.

Unexpectedly, males in our sample demonstrated less %TGW gain than females at 3 and 6 months after starting FBT, despite starting treatment at a commensurate %TGW and having similar rates of weight restoration. Weight-related treatment outcomes in males with EDs are mixed, including similar [47, 48], better [39], and worse [49] outcomes compared to females. Our findings suggest that males may require a longer duration of treatment to achieve weight restoration. This could reflect lower perceptions of treatment urgency in caregivers of males with AN that could be secondary to well-documented treatment barriers and misconceptions about EDs in males [49].

We also found that adolescents who started outpatient FBT at a higher %TGW showed less %TGW gain at 3 and 6 months. However, these patients were more likely to achieve full weight restoration (i.e., ≥ 95% of TGW) than patients who started treatment at a lower TGW. Patients who were medically hospitalized in our sample before starting FBT demonstrated significantly greater %TGW gain in the first 3 months of outpatient FBT compared to patients who did not require hospitalization. This could reflect the unique provision of a brief FBT-guided intervention to caregivers of hospitalized patients, prior to starting standard outpatient FBT. Whereas this is a methodological limitation of our study, it did not impact our study aim, insofar as pre-treatment caregiver burden was not associated with %TGW gain at 3 and 6 months in our sample when controlling for hospitalization status. It is also notable that participants completed all questionnaires before any FBT-guided interventions were provided, so responses were not differentially biased by inconsistent exposure to FBT principles. Finally, 6 months after starting FBT, hospitalized patients no longer demonstrated greater %TGW gain, suggesting similar weight gain trajectories for patients with or without initial hospitalizations before starting FBT over time.

## Strengths and limits

Our study is one of few to examine and identify predictors of caregiver burden in a large sample of adolescents with AN/AAN before starting FBT. To our knowledge, it is the first to examine associations between pre-treatment FBT and %TGW gain throughout treatment. Potential limitations include our singular assessment of caregiver burden and our mixed sample of patients with and without medical admissions before starting treatment. Further, caregiver burden scores in our sample (EDSIS mean score = 31.3, SD = 12.7) were lower than those reported in ED caregivers of adults with AN (*n* = 67; EDSIS mean score = 41.0, SD = 12.6) [28] and adolescents with AN (*n* = 50; EDSIS mean score = 65.7, SD = 11.9) [17]. This discrepancy could reflect our larger sample size, which potentially yielded a more normalized and representative distribution of EDSIS scores [50]. Our study was also limited to a particularly homogenous sample, with very little racial and gender diversity. It is well documented that EDs do not discriminate among minority groups [51, 52]. Our findings must be interpreted in the context of mothers and mostly female, straight, and White adolescents with AN and access to health care.

## Conclusions

Caregivers play a central role in FBT and are instrumental to an adolescent’s recovery from AN. Our study suggests that caregiver anxiety, family history of EDs, adolescent history of mental health treatment, and ED symptom severity places caregivers at risk for greater caregiver burden before starting treatment. Proactive assessment of caregiver burden and associated vulnerabilities, coupled with targeted interventions to ameliorate identified caregiver difficulties (e.g., facilitating mental health referrals, prioritizing social support for caregivers) are suggested. Our findings also provide preliminary support that males may be susceptible to slower weight-related progress in FBT and consequently, longer treatment durations. Thus, increased vigilance to and prioritization of rapid weight gain among males may be warranted to maximize treatment success.

### What is already known on this subject?

Caregiver burden is common among caregivers of individuals with eating disorders (EDs). In family-based treatment (FBT) for anorexia nervosa (AN), caregiver beliefs and behaviors are associated with treatment outcomes.

### What does this study add?

Caregiver burden was predicted by caregiver anxiety, family history of EDs, adolescent history of mental health treatment, and ED symptom severity before starting FBT. Males made slower progress toward weight restoration at 3 and 6 months after starting treatment, suggesting longer courses of FBT.

## Data Availability

The datasets generated and/or analyzed during the current study are available from the corresponding author on reasonable request.
